# Mesenteric Cyst: A Rare Entity

**DOI:** 10.7759/cureus.48963

**Published:** 2023-11-17

**Authors:** Shailja Singh, Rushikesh K Shukla, Pankaj Gharde

**Affiliations:** 1 Department of Surgery, Jawaharlal Nehru Medical College, Datta Meghe Institute of Higher Education and Research, Wardha, IND

**Keywords:** small bowl mesentery, laprotomy, gi cyst, gi surgery, mesenteric cyst

## Abstract

Mesenteric cysts are rare entities that are challenging to diagnose and treat because of their variable presentation and histological characteristics. They have been majorly classified into six groups, out of which, the chylo-lymphatic type is the most common. Their etiology remains poorly understood but is usually linked to lymphatic pathologies. They are thin-walled cysts, present in the mesentery of the gastrointestinal tract. They can mimic multiple other cysts; hence, their timely diagnosis is of utmost importance. Imaging techniques aid in the preoperative diagnosis along with a thorough physical exam. The mainstay of treatment is surgical excision of the cyst, which is essential to prevent the recurrence of malignant transformation; the usual method of removal is laparoscopy. Alternative treatments are aspiration and marsupialization, which are only utilised for specific cases. The recurrence rate is usually low after total excision, but follow-ups are recommended for early detection of recurrence. This case study highlights the significance of prompt diagnosis and proper management of mesenteric cysts.

## Introduction

The term mesenteric cyst refers to a diverse set of cystic lesions with various origins that manifest in the retroperitoneum or abdomen. Italian anatomist Benevieni first discovered it in 1507 while conducting an autopsy on an eight-year-old. In 1880, Tillaux performed its first surgical excision [1]. Females outnumber males in a ratio of 2:1 in these uncommon, benign intraabdominal tumours, which have reported incidences of 1/100,000 in adults and 1/20,000 in infants [2]. These cysts can form at any place in the gastrointestinal tract’s mesentery, from the rectum to the duodenum. They might extend into the retroperitoneum from the mesentery's base. Sixty percent of mesenteric cysts in a group of 162 patients were found in the small intestine, whereas twenty percent were found in the large intestine mesentery, and fifteen percent in the retroperitoneum [3]. They can present as multiple or simple cysts, can contain serous, haemorrhagic, purulent, or chylous fluid, and can also be unilocular or multilocular [4,5]. Upon histopathological analysis of the surgical specimen, a single layer of flattened mesothelial immunoreactive cells with cytokeratins and a fibrous wall with lymphocytes may be found surrounding a unilocular or multilocular cyst [1]. Although uncommon, they do carry a 3% chance of developing malignant transformation. They typically exhibit a wide range of vague symptoms, and 40% of cases are discovered accidentally during routine general physical exams or imaging techniques. Although the cause of mesenteric cysts is unknown, a prevalent theory suggests that lymph nodes may not be able to interact with the lymphatic or venous systems or that the lymphatic system may be blocked [6].

## Case presentation

A four-year-old male child came with chief complaints of pain abdomen for the past four days. According to the mother's account, the child was all right four days back when he developed pain in the abdomen, which was acute in onset, sharp and diffuse in nature, non-radiating, and with no associated vomiting, nausea, or diarrhoea. The patient had a history of black-coloured stools for the last two days. The child was not accepting well orally well since four days. There was no significant past medical or surgical history. The general physical and systemic examinations were normal. All the required blood investigations were done. Table 1 shows the findings of complete blood count investigations with peripheral smear.

**Table 1:** Complete blood count investigations on cell counter with peripheral smear Hb: Haemoglobin; HCT: Haematocrit; MCHC: Mean corpuscular haemoglobin concentration; MCV: Mean corpuscular volume; MCH: Mean corpuscular haemoglobin; RDW: Red cell distribution width

Hb%	Total RBC count	Total WBC count	HCT	MCHC	MCV	MCH	Total platelet count	Monocytes	Granulocytes	Lymphocytes	RDW	Eosinophils	Basophils
10.6	4.66	5800	32.3	32.9	69.3	22.8	9.93	04	55	40	17.3	01	00
Peripheral smear													
RBCs- Normocytic normochromic; Platelets- Adequate on smear. No haemoparasite seen													

Table 2 shows the findings of the coagulation profile of the patient.

**Table 2:** Coagulation profile - APTT, PT APTT: Activated partial thromboplastin time; PT: Prothrombin time; INR: International normalised ratio

APTT - control	APTT - patient	PT - control	PT - patient	INR
29.5	29.7	11.9	11.9	1.00

The following Table 3 and Table 4 show the liver function tests and kidney function tests of the patient, respectively.

**Table 3:** Liver function tests of the patient LFT: Liver function test; ALT (SGPT): Alanine aminotransferase (serum glutamic pyruvic transaminase); AST (SGOT): Aspartate aminotransferase (serum glutamic oxaloacetic transaminase); BC: Bilirubin conjugated; BU: Bilirubin unconjugated

LFT								
Alkaline phosphatase	ALT (SGPT)	AST (SGOT)	Total Protein	Albumin	Total Bilirubin	BC	BU	Globulin (calculated parameter)
141	15	34	6.5	3.6	0.5	0.2	0.3	2.9

**Table 4:** Kidney function tests of the patient KFT: Kidney function test

KFT			
Urea	Creatinine	Sodium (Na+)	Potassium (K+)
29	0.4	142	4.4

**Figure 1:**
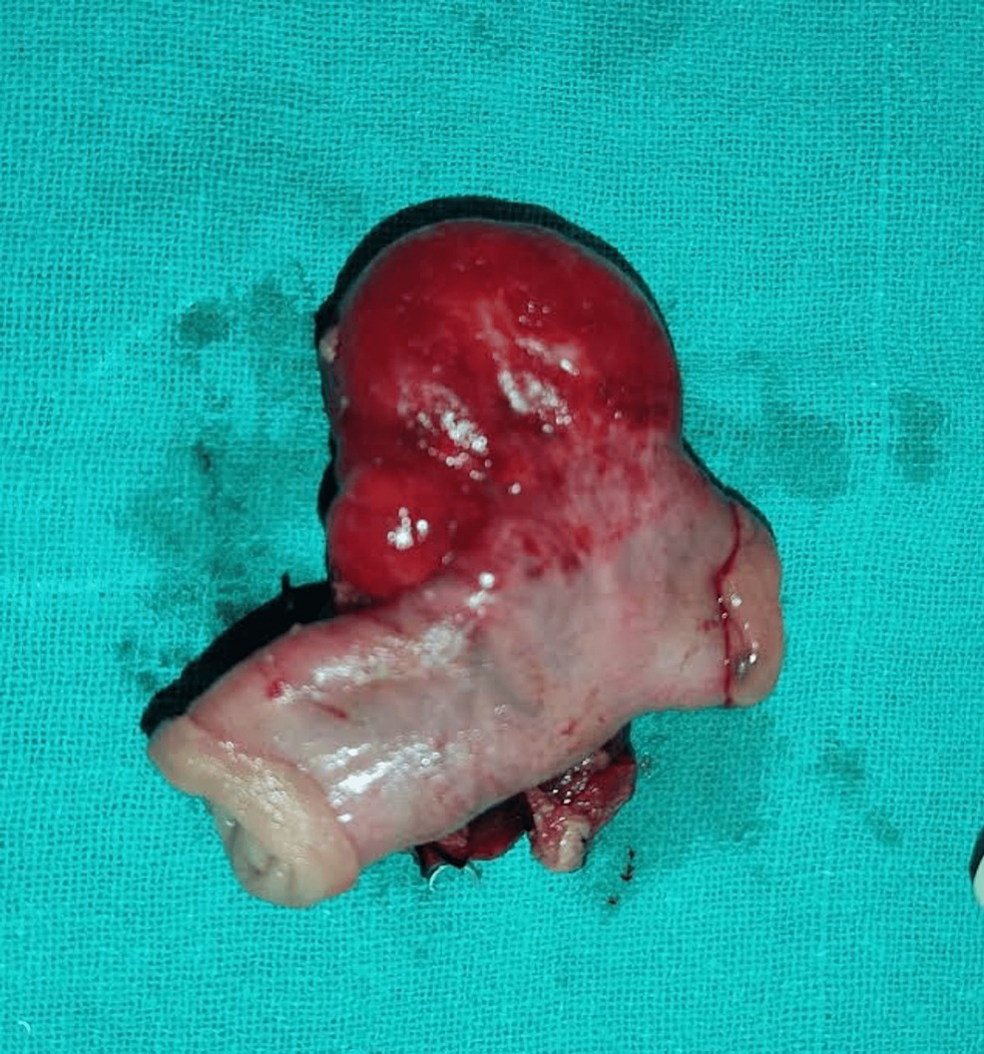
Resected mesenteric cyst with adherent small bowel

Ultrasound abdomen and pelvis was done which showed a complex cystic lesion in the abdomen and the patient was further advised for a CT scan. CT scan showed evidence of a mesenteric cyst with left-sided mild hydronephrosis with pelvic ureteric junction obstruction and minimal free fluid in the peritoneal cavity. The patient was advised exploratory laparotomy. One unit of packed red cell transfusion was administered. The child was orally allowed and started on intravenous fluids, injection ceftriaxone 1 gram twice daily, pantoprazole 40 mg once daily, ondansetron 4 mg once daily, and tramadol 50 mg in 100 ml normal saline intravenous. The patient underwent laparotomy for the excision of the mesenteric cyst with adherent small bowel resection and anastomosis (Figure 1). 

**Figure 2:**
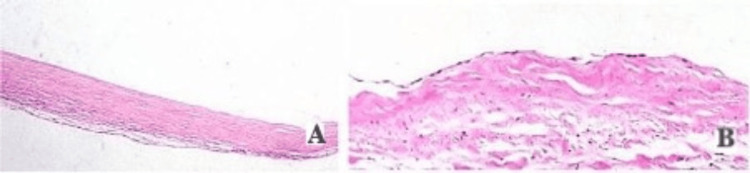
Histopathological diagram consistent with mesenteric cyst showing fibrocollagenous tissue, adipose tissue, and chronic inflammatory infiltrates

Intraoperatively dilated small mass with enlarged mesenteric lymph nodes and cystic lesion adherent to jejunum were noted about 50 cm from duodeno-jejunal flexure. A mesenteric lymph node biopsy was taken and was sent for histopathology. Postoperatively the specimen was examined and it was suggestive of fibrocollagenous tissue, adipose tissue, and chronic inflammatory infiltrate. Figure 2 shows the histopathology showing the above-mentioned characteristics.

Postoperatively the patient was kept nil by mouth, one unit of packed red cell transfusion was administered and started on intravenous fluids, injection ceftriaxone, injection metronidazole, and injection amikacin. The patient's postoperative period was uneventful, and he was discharged.

## Discussion

The majority of mesenteric cysts are benign lesions, although they frequently appear with a variety of nonspecific symptoms, including nausea, vomiting, anorexia, stomach pain, and changes in bowel patterns [3,6]. Palpable masses, obstructive uropathies, and intestinal obstructions are possible in advanced stages [7]. Other emergency scenarios include the cyst rupturing into the peritoneum and causing peritonitis, volvulus, ileus, infection, perforation, and haemorrhage [8]. Rarely, the clinical presentation may be dramatic, with significant abdominal pain that is immediate and intense, showing signs of intestinal blockage, or mimicking the rupture of an aortic aneurysm [9]. The differential diagnosis of mesenteric cyst includes numerous other cysts like gastrointestinal duplication, paratubal, pancreatic, ovarian, adrenal, choledochal, splenic, renal, omental, hydatid, omphalomesenteric duct, urachal cyst, and other pathologies like hydronephrosis, and ascites [10], which can be distinguished with a complete radiological investigation, physical examination, and history collection. The fluid inside the cyst may have a variety of properties, including being hemorrhagic, serous, chylous, or polluted [7]. 

In 2000, de Perrot et al. [2] developed a categorization that is now the most often used, which divided the mesenteric cysts into six groups based on histological characteristics: mature cyst teratoma, lymphatic cyst, urogenital cyst, mesothelial cyst, enteric cyst, and pseudocyst [11]. The most prevalent of these is the chylo-lymphatic type, which is believed to result from abnormal lymphatic development without efferent communication, obstruction of lymph ducts, degeneration of lymphatics, proliferation of ectopic lymphoid tissues, injury to lymph ducts, degeneration of lymph nodes, and failure of fusion of mesenteric leaves; however, these theories have not yet been proven [8]. These are frequently isolated, thin-walled, and unilocular in the ileal mesentery [7]. The formation of the developing mesentery is assumed to be the cause of the enterogenous variety, which is another variation.

According to their genesis and histological characteristics, mesenteric cysts can also be divided into four different categories: (1) fetal and developmental cysts; (2) infectious or degenerative cysts; (3) traumatic or acquired cysts; and (4) neoplastic cysts. Neoplastic cysts and fetal and developmental cysts are examples of real cysts that are created by endothelial cells. False cysts with an inflammatory cell-lined fibrous cystic wall include traumatic cysts, infectious and degenerative cysts, and other cysts [8]. Mesenteric cysts may resemble disorders including aortic aneurysms, pancreatic pseudocysts, cystic tumours, and pelvic ailments, making it difficult to diagnose them [3]. Due to the numerous problems associated with delayed diagnosis, which can result in suboptimal surgical outcomes, early preoperative detection of these mesenteric cysts is essential in determining the therapeutic strategy [2]. With the help of routine abdominal imaging tests (ultrasonography, CT, nuclear magnetic resonance), a preoperative diagnosis can be formed.

**Figure 3:**
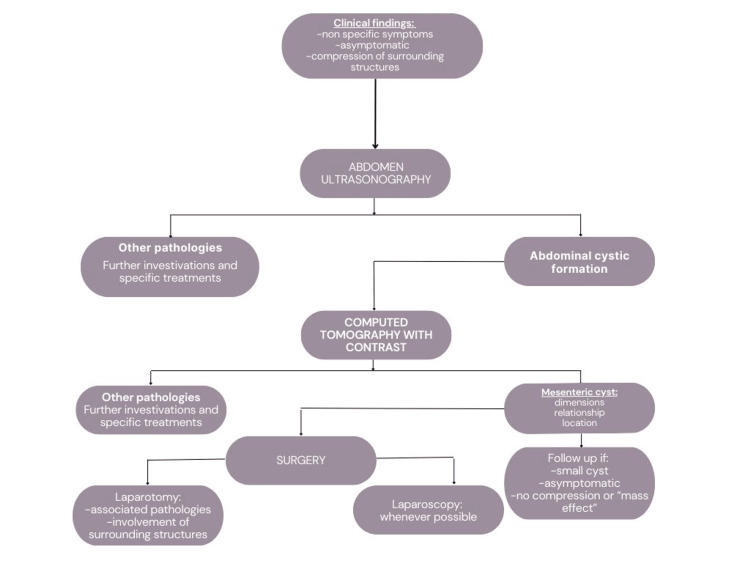
Management approach Image Credit: Dioscoridi et al., 2014 [12]; Licensed under CC BY-NC 3.0

A cystic tumour is typically visible on ultrasound, and its content may create a fluid-fluid level. A cystic tumour with a thick wall and fluid content with a low CT number is seen on CT imaging [9]. A CT scan of the abdomen can assist in quantifying the mass's size more precisely, defining its extent, illustrating how it relates to other abdominal organs, identifying calcium deposits, and determining whether or not there is a retroperitoneal extension [10]. A comprehensive clinical examination, a complete medical history, all regular blood tests, and radiographic examinations are recommended in instances of suspected mesenteric cysts in order to make a preliminary diagnosis [7]. Most patients will require a laparoscopy to confirm the diagnosis because it is challenging to differentiate between these tumours on ultrasonography. As ovarian torsion or acute appendicitis are two conditions that may mimic cyst torsion and need immediate exploration, this can happen [10]. The following Figure 3 shows a rough outline of the management approach regarding the treatment of mesenteric cysts.

The first-choice treatment is complete excision via surgery, which may necessitate removing a portion of the mesentery along with the tumour so as to prevent recurrence and the potential danger of malignant transformation. Laparotomy or laparoscopy might be used to remove the cyst [6]. Simple aspiration or marsupialization may be considered when short bowel syndrome is predicted but is typically not advised due to infection and frequent recurrence [8]. If enucleation cannot be performed safely due to the cyst wall adhering to the mesenteric tissue and other tissues nearby, a resection of the neighbouring organ may be necessary. Other treatments include de-roofing, drainage, and partial cyst excision. Because of their higher risk of recurrence, these treatments are rarely suggested. Secondary issues associated with mesenteric cysts include volvulus, infectious fluid leaking, herniation of the colon into an abdominal defect, and obstruction [7]. It is not recommended to leave a cyst untreated because, despite being extremely rare, a malignant change has been documented. However, due to some cysts' multiloculated nature, intraoperative rupture might result in insufficient excision and, ultimately, recurrence.

Some little cysts might potentially go unnoticed, growing larger and leading to a recurrence. The rate of recurrence is typically modest (0-15%) and significantly lower following total surgical excision [10]. For such patients, routine follow-ups with an abdomen ultrasound are indicated in order to detect the mesenteric cyst's recurrence early and lower the morbidity. CT-guided aspiration is recommended in the event that the cyst reappears at any point [7]. The patient's course of therapy and the result described in this case study seem to be in line with the accepted wisdom on the management of mesenteric cysts and related issues. A frequent surgical intervention for this disease is the choice to proceed with an exploratory laparotomy, which includes the removal of the mesenteric cyst and resection of adherent small bowel with subsequent anastomosis. The patient's result matched comparable case studies very well [1,6,7].

## Conclusions

A four-year-old child presented with complaints of severe stomach ache, dark stool, and inadequate oral intake. A mesenteric cyst with related kidney and ureter pathology was discovered by imaging. He underwent a laparotomy, which included lymph node biopsy, small intestinal resection, and cyst removal. He was administered antibiotics and fluid after the surgery; he healed well and was eventually discharged. In summary, mesenteric cysts are interesting entities that require a multidisciplinary approach involving multiple departments like radiology, surgery, and pathology for early diagnosis and optimal treatment. Even though they are uncommon, they have the potential for complications, which justifies the significance of early intervention and continuous monitoring, which ensures the most favourable outcome for patients with mesenteric cysts. Technological advances in medical imaging and surgical techniques continue to air and improve our ability to diagnose and treat these unique tumours. A regular follow-up schedule is essential for patients who have undergone surgery, as there is a risk of recurrence. Early detection of recurrence through various modalities can help manage these cases effectively.
